# Pharmacovigilance-based 12-year post-marketing safety analysis of quadrivalent influenza vaccines in children: a VAERS surveillance study

**DOI:** 10.3389/fped.2025.1640498

**Published:** 2025-09-02

**Authors:** Honghong Fu, Yirong Huang, Xinjin Wang, Ying Zhang, Yijie Liu, Yonglong Su, Lili Pan

**Affiliations:** ^1^ Department of Biological Products Testing, Fujian Institute for Food and Drug Quality Control, Fuzhou, Fujian, China; ^2^ Department of Pharmacy, Fujian Medical University Union Hospital, Fuzhou, Fujian, China; ^3^ Department of Business Management Section, Fujian Center For Drug Inspection, Fuzhou, Fujian, China; ^4^ Department of Clinical Pharmacy, Xiamen Haicang Hospital, Xiamen, Fujian, China; ^5^ Fujian Institute of Hematology, Fujian Provincial Key Laboratory on Hematology, Fujian Medical University Union Hospital, Fuzhou, Fujian, China

**Keywords:** quadrivalent influenza vaccine, pediatric safety, adverse events, VAERS, Guillain–Barré syndrome, post-marketing surveillance

## Abstract

**Background:**

Quadrivalent influenza vaccines (QIVs) are extensively administered to children for seasonal influenza prevention. However, comprehensive long-term evaluations of post-marketing safety remain limited.

**Methods:**

We conducted a comprehensive 12-year pharmacovigilance analysis utilizing data from the Vaccine Adverse Event Reporting System (VAERS) for individuals under 18 years receiving six FDA-approved QIVs between 2013 and 2024, representing three distinct platforms: four egg-based inactivated vaccines (Afluria, Fluarix, FluLaval, Fluzone), one cell culture-based inactivated vaccine (Flucelvax), and one live-attenuated intranasal vaccine (FluMist). Disproportionality analyses using reporting odds ratios (ROR) and Bayesian information component (IC) were employed to identify safety signals. Safety signals were considered statistically significant when the lower bound of the 95% confidence interval for ROR exceeded 1.0 and the lower bound of the IC (IC025) exceeded 0.

**Results:**

Of the 15,458 reported adverse events, 5.29% (95% CI: 4.95–5.65) were classified as serious, including 67 fatalities. Guillain-Barré syndrome (GBS) emerged as a statistically significant safety signal (42 cases; ROR = 1.71, 95% CI: 1.25–2.35; IC025 = 0.28). Among specific QIV products, Flucelvax demonstrated the lowest proportion of serious adverse events (2.35%). Notably, reporting volumes decreased by 42% during the COVID-19 pandemic.

**Conclusion:**

This pharmacovigilance analysis demonstrates generally favorable safety profiles for pediatric QIVs, with cell culture formulations showing superior safety characteristics. The extremely low absolute risk of Guillain-Barré syndrome supports continued routine pediatric influenza vaccination while highlighting the importance of platform-specific safety monitoring.

## 1 Introduction

Annual influenza vaccination remains a cornerstone of pediatric preventive healthcare, with quadrivalent influenza vaccines (QIVs) now established as the global standard of care (1). The transition from trivalent to quadrivalent formulations between 2012 and 2015 aimed to expand immunological coverage by incorporating both influenza B virus lineages, thereby enhancing population-level protection (2, 3). Currently, six QIVs approved by the U.S. Food and Drug Administration (FDA) are available for pediatric use, including egg-based and cell culture-derived platforms, as well as live attenuated and inactivated vaccine formulations (4).

Although pre-licensure clinical trials are critical for establishing initial vaccine safety and immunogenicity profiles, they typically lack the statistical power to detect rare adverse events. Consequently, comprehensive post-marketing surveillance remains essential for monitoring vaccine safety (5). In the United States, the Vaccine Adverse Event Reporting System (VAERS), co-managed by the Centers for Disease Control and Prevention (CDC) and the FDA since 1990, serves as the principal passive surveillance platform for monitoring vaccine-related adverse events (6).

Previous pediatric influenza vaccine safety studies using VAERS have primarily focused on specific safety concerns or limited time periods. Notably, Vellozzi et al. examined VAERS reports for 2009 H1N1 monovalent vaccines, while studies such as those by McMahon et al. analyzed adverse events following inactivated influenza vaccination in children under 2 years using VAERS data from 1990 to 2003 (7, 8). More recent Vaccine Safety Datalink (VSD) studies by Tse et al. and Duffy et al. concentrated on febrile seizures following trivalent influenza vaccines in children aged 6–23 months (9, 10). However, these studies have several limitations: they either predate the widespread adoption of QIVs, focus on specific adverse events rather than comprehensive safety profiles, or examine limited age ranges or time periods. Our study uniquely provides the first comprehensive, longitudinal analysis spanning the entire post-QIV implementation period (2012–2024), comparing safety profiles across all FDA-approved pediatric QIV formulations using standardized methodology.

The heterogeneity among QIV platforms necessitates platform-specific safety evaluation due to fundamental differences in manufacturing processes, antigen presentation, and immune activation patterns. Egg-based vaccines may contain residual egg proteins and ovalbumin that could trigger allergic reactions in sensitized individuals, despite extensive purification processes (11, 12). Cell culture-derived vaccines eliminate egg-related allergens and avoid potential egg-adaptation mutations that may affect immunogenicity, but may present different glycosylation patterns due to mammalian cell production systems (13, 14). Live-attenuated influenza vaccines (LAIV) employ temperature-sensitive, replication-competent viruses that replicate in the nasopharynx and may cause distinct adverse event patterns, including potential concerns about viral shedding and transmission risks to immunocompromised contacts (15, 16). Additionally, the different manufacturing processes, adjuvant systems, and preservatives used across platforms may contribute to varying reactogenicity profiles. Given these biological and manufacturing differences, platform-specific safety analysis is essential for identify potential differential risk signals that could inform clinical decision-making and regulatory guidance.

Guillain-Barré syndrome (GBS) has been consistently recognized as a rare but notable adverse event following influenza vaccination in adult populations, with an estimated excess risk ranging from 1 to 2 cases per million doses administered (17, 18). However, pediatric-specific GBS risk estimates are more limited and suggest potentially different risk profiles. Studies suggest that pediatric GBS following influenza vaccination occurs at lower rates than in adults, though precise estimates vary by study methodology and population (19, 20). Additionally, background GBS incidence rates differ substantially by age, with pediatric populations showing incidence rates of 0.62–0.69 per 100,000 person-years compared to higher adult rates that increase with age (21, 22). Despite these lower baseline rates, detailed characterization of GBS risk across different QIV formulations in pediatric populations remains limited, highlighting the need for platform-specific, pediatric surveillance data.

Additionally, the COVID-19 pandemic has disrupted routine pediatric immunization practices and healthcare utilization, potentially influencing both the frequency of vaccine-associated adverse events and their reporting patterns (23, 24).

This study provides a 12-year comprehensive analysis of post-marketing safety for all FDA-approved pediatric QIVs by leveraging data from VAERS. Our aim is to deliver a detailed comparative safety profile and address critical gaps in vaccine pharmacovigilance, examining both pre-pandemic and pandemic reporting periods.

## 2 Materials and methods

### 2.1 Data source and extraction

Adverse event reports were retrieved from the Vaccine Adverse Event Reporting System (VAERS) via the CDC WONDER interface, covering the period from January 1, 2013–December 31, 2024 (accessed March 2025). The dataset included patient demographics, vaccine identifiers, Medical Dictionary for Regulatory Activities (MedDRA) preferred terms, and serious event indicators (6).

### 2.2 Study population, inclusion criteria, and data handling

The study included reports for individuals younger than 18 years of age where an FDA-approved quadrivalent influenza vaccine (QIV) was coded as the primary suspected vaccine. Reports were systematically excluded if they (i) lacked age information, (ii) did not specify the QIV formulation, (iii) were flagged as duplicates through an automated pipeline (collapsing records with identical VAERS_ID and retaining only the most recent RECVDATE), or (iv) were incomplete, defined as lacking both vaccine type and any coded MedDRA preferred term. Data cleaning and handling of missing values were automated using scripted rules in R without manual adjudication or imputation; analyses requiring specific variables (e.g., sex, onset interval) were performed on complete-case subsets.

### 2.3 Vaccine classification and characteristics

Six FDA-approved QIVs were evaluated:

#### 2.3.1 Egg-based inactivated vaccines

Afluria Quadrivalent (CSL Seqirus; approved 2016).

Fluarix Quadrivalent (GSK; approved 2012).

FluLaval Quadrivalent (GSK; approved 2013).

Fluzone Quadrivalent (Sanofi Pasteur; approved 2013).

#### 2.3.2 Cell culture-based inactivated vaccine

Flucelvax Quadrivalent (CSL Seqirus; approved 2016).

#### 2.3.3 Live attenuated intranasal vaccine

FluMist Quadrivalent (AstraZeneca; approved 2012).

### 2.4 Outcome definitions

Serious adverse events (SAEs) were defined according to FDA criteria, including death, life-threatening conditions, hospitalization or prolonged hospitalization, persistent disability, and congenital anomalies (25). All adverse events were coded using MedDRA (version 27.0).

### 2.5 Statistical analysis

Categorical variables were summarized as frequencies and proportions, and continuous variables as medians with interquartile ranges (IQRs). Age was stratified into four groups: younger than 6 months, 6 months–5 years, 6–12 years, and 13–17 years.

Disproportionality analyses employed two complementary metrics: (i) reporting odds ratio (ROR), calculated as ROR = (a × d)/(b × c), where a = reports with the event and vaccine of interest, b = other events with the vaccine of interest, c = the event with other vaccines, and d = other events with other vaccines; and (ii) information component (IC), an empirical Bayesian shrinkage measure robust for sparse data, with IC025 indicating the lower 95% credibility bound. A safety signal was identified when both the lower 95% confidence interval of the ROR exceeded 1.0 and IC025>0. To account for multiple comparisons across MedDRA preferred terms, *p*-values from ROR analyses were adjusted using the Benjamini-Hochberg false discovery rate (FDR) procedure (q < 0.05) (26). All analyses were conducted using R statistical software (version 4.4.2).

### 2.6 Ethical statement

This study utilized publicly available and de-identified VAERS data; therefore, institutional review board (IRB) approval was not required. The analysis was conducted in accordance with the principles of the Declaration of Helsinki.

## 3 Results

### 3.1 Overall report characteristics

A total of 15,458 pediatric adverse event reports were included, with a median patient age of 7.3 years [interquartile range (IQR): 2.8–11.5 years]. The gender distribution was balanced, with females representing 47.5% of cases. Vaccine-specific report distribution was as follows: Fluzone (39.9%, *n* = 6,166), Fluarix (16.8%, *n* = 2,596), FluLaval (16.7%, *n* = 2,581), FluMist (15.4%, *n* = 2,373), Flucelvax (7.7%, *n* = 1,192), and Afluria (3.6%, *n* = 550).

### 3.2 Serious adverse events and mortality

Serious adverse events (SAEs) were reported in 818 cases (5.29%). The SAE proportions by vaccine were highest for Afluria (6.73%), followed by Fluarix (6.32%), FluMist (5.77%), Fluzone (5.71%), FluLaval (3.87%), and Flucelvax, which exhibited the lowest SAE rate (2.35%).

There were 67 reported deaths, corresponding to a crude mortality rate of 0.43%. Notably, 73% of fatalities occurred among children aged 6 months to 5 years, with 54% involving Fluzone. These associations are temporal, and causality should not be inferred from these reports given the limitations of passive surveillance data (27).

### 3.3 Age-Stratified safety patterns

An inverse relationship between age and SAE frequency was observed across all vaccines. Among children aged 6 months to 5 years, SAE rates ranged from 2.37% (Flucelvax) to 10.16% (Afluria). Adolescents aged 13–17 years showed substantially lower SAE rates, ranging from 1.78% to 6.73%. Table 1 presents the detailed age-specific distribution of serious adverse events following quadrivalent influenza vaccination.

**Table 1:** Age-specific distribution of serious adverse events (SAEs) following quadrivalent influenza vaccination in children aged <18 years (2013–2024).

Vaccine name (Total, *n*)	Age distribution, *n* (%)		Sex distribution, *n* (%)		SAEs, *n* (Deaths)	SAE rate (95% CI)
AFLURIA (550)	13–17 years	154 (28%)	F	89 (16.18%)	6	6.73% (4.78%–9.15%)
			M	64 (11.64%)	2	
			U	1 (0.18%)	0	
	6–12 years	201 (36.55%)	F	101 (18.36%)	6	
			M	100 (18.18%)	4	
	6 months–5 years	187 (34%)	F	81 (14.73%)	9 (1)	
			M	105 (19.09%)	10	
			U	1 (0.18%)	0	
	<6 months	8 (1.45%)	F	5 (0.91%)	0	
			M	3 (0.55%)	0	
FLUARIX (2,596)	13–17 years	618 (23.81%)	F	335 (12.9%)	17	6.32% (5.41%–7.32%)
			M	277 (10.67%)	14	
			U	6 (0.23%)	0	
	6–12 years	982 (37.83%)	F	451 (17.37%)	28 (1)	
			M	514 (19.8%)	18 (3)	
			U	17 (0.65%)	0	
	6 months–5 years	963 (37.1%)	F	443 (17.06%)	32	
			M	498 (19.18%)	53 (4)	
			U	22 (0.85%)	1	
	<6 months	33 (1.27%)	F	15 (0.58%)	0	
			M	17 (0.65%)	1	
			U	1 (0.04%)	0	
FLULAVAL (2,581)	13–17 years	460 (17.82%)	F	257 (9.96%)	7 (1)	3.87% (3.16%–4.69%)
			M	198 (7.67%)	6 (1)	
			U	5 (0.19%)	0	
	6–12 years	924 (35.8%)	F	440 (17.05%)	20 (1)	
			M	476 (18.44%)	9	
			U	8 (0.31%)	0	
	6 months–5 years	1,133 (43.9%)	F	493 (19.1%)	21 (3)	
			M	617 (23.91%)	37 (4)	
			U	23 (0.89%)	0	
	<6 months	64 (2.48%)	F	27 (1.05%)	0	
			M	32 (1.24%)	0	
			U	5 (0.19%)	0	
FLUCELVAX (1,192)	13–17 years	384 (32.21%)	F	233 (19.55%)	7	2.35% (1.57%–3.38%)
			M	141 (11.83%)	5 (1)	
			U	10 (0.84%)	0	
	6–12 years	507 (42.53%)	F	270 (22.65%)	2	
			M	234 (19.63%)	7	
			U	3 (0.25%)	0	
	6 months–5 years	295 (24.75%)	F	128 (10.74%)	4 (1)	
			M	160 (13.42%)	3	
			U	7 (0.59%)	0	
	<6 months	6 (0.5%)	F	2 (0.17%)	0	
			M	4 (0.34%)	0	
FLUMIST (2,373)	13–17 years	340 (14.33%)	F	176 (7.42%)	9 (3)	5.86% (4.95%–6.88%)
			M	162 (6.83%)	10	
			U	2 (0.08%)	0	
	6–12 years	998 (42.06%)	F	490 (20.65%)	35 (3)	
			M	489 (20.61%)	19	
			U	19 (0.8%)	0	
	2–5 years	880 (37.08%)	F	419 (17.66%)	21 (1)	
			M	436 (18.37%)	41 (3)	
			U	25 (1.05%)	2	
	6 months–1 years	144 (6.07%)	F	67 (2.82%)	0	
			M	63 (2.65%)	2	
			U	14 (0.59%)	0	
	<6 months	11 (0.46%)	F	2 (0.08%)	0	
			M	7 (0.29%)	0	
			U	2 (0.08%)	0	
FLUZONE (6,166)	13–17 years	1,021 (16.56%)	F	539 (8.74%)	21	5.76% (5.19%–6.37%)
			M	463 (7.51%)	33 (1)	
			U	19 (0.31%)	0	
	6–12 years	1,942 (31.5%)	F	895 (14.52%)	29 (2)	
			M	996 (16.15%)	30 (1)	
			U	51 (0.83%)	0	
	6 months–5 years	3,019 (48.96%)	F	1,307 (21.2%)	99 (11)	
			M	1,604 (26.01%)	139 (20)	
			U	108 (1.75%)	3 (1)	
	<6 months	184 (2.98%)	F	85 (1.38%)	1	
			M	76 (1.23%)	0	
			U	23 (0.37%)	0	

This table presents the number and percentage of serious adverse events (SAEs) reported to the Vaccine Adverse Event Reporting System (VAERS) between 2013 and 2024, stratified by vaccine type, age group and sex in children under 18 years of age. SAE rates are presented for each vaccine formulation with corresponding 95% confidence intervals (CIs), calculated using the Clopper–Pearson exact method. The denominator (*n*) in each case refers to the number of reports submitted for the corresponding vaccine. SAEs were defined according to U.S. FDA criteria and include hospitalization, life-threatening illness, permanent disability, and death. The highest SAE proportions were consistently observed among children aged 6 months to 5 years. Data were obtained through passive surveillance; therefore, they are subject to underreporting and reporting bias.

### 3.4 Disproportionality signal detection

A total of 251 MedDRA preferred terms met predefined criteria for disproportionality signals across all QIVs. Notable vaccine-specific safety signals included syncope and seizures associated with Afluria, injection-site reactions linked primarily to egg-based vaccines, and respiratory symptoms observed with FluMist.

Among evaluated rare adverse events, Guillain-Barré syndrome (GBS) was the sole condition meeting both statistical thresholds, with 42 reported cases (ROR = 1.71, 95% CI: 1.25–2.35; IC025 = 0.28). The median time to symptom onset for GBS was 14.5 days (IQR: 4.5–32 days), with distribution across vaccines as follows: Fluzone (*n* = 16), Fluarix (*n* = 7), FluMist (*n* = 6), FluLaval (*n* = 4), Afluria (*n* = 3), and Flucelvax (*n* = 0). Table 2 presents the top five MedDRA preferred terms showing positive disproportionality signals, while Table 3 provides a comprehensive summary of rare adverse events.

**Table 2:** Top five MedDRA preferred terms showing positive disproportionality signals associated with quadrivalent influenza vaccines in pediatric populations.

Vaccine name	Preferred term	SOC (MedDRA)	ROR (95% CI)	IC (IC025)
AFLURIA	Seizure	Nervous system disorders	4.37 (3.13–6.11)	2.10 (1.61)
	Fall	Injury & procedural complications	3.56 (2.43–5.21)	1.81 (1.26)
	Loss of consciousness	Nervous system disorders	3.65 (2.79–4.78)	1.83 (1.44)
	Syncope	Nervous system disorders	3.59 (2.88–4.47)	1.80 (1.48)
	Dizziness	Nervous system disorders	2.28 (1.76–2.96)	1.17 (0.79)
FLUARIX	Peripheral swelling	General disorders & administration site conditions	4.87 (3.96–5.99)	2.23 (1.93)
	Skin warm	Skin & subcutaneous tissue disorders	3.00 (2.49–3.61)	1.55 (1.28)
	Fall	Injury & procedural complications	2.89 (2.34–3.56)	1.50 (1.20)
	Injection-site warmth	General disorders & administration site conditions	2.49 (2.16–2.86)	1.28 (1.07)
	Injection site pain	General disorders & administration site conditions	2.27 (1.97–2.62)	1.13 (0.93)
FLUCELVAX	Loss of consciousness	Nervous system disorders	5.35 (4.52–6.34)	2.36 (2.11)
	Fall	Injury & procedural complications	5.32 (4.20–6.75)	2.37 (2.03)
	Syncope	Nervous system disorders	4.89 (4.24–5.65)	2.21 (2.00)
	Unresponsive to stimuli	Nervous system disorders	4.45 (3.40–5.82)	2.12 (1.73)
	Dizziness	Nervous system disorders	3.08 (2.60–3.65)	1.58 (1.33)
FLULAVAL	Peripheral swelling	General disorders & administration site conditions	5.15 (4.18–6.35)	2.31 (2.01)
	Extra dose administered	Injury & procedural complications	4.11 (3.43–4.93)	1.99 (1.73)
	Product storage error	Injury & procedural complications	3.51 (3.11–3.95)	1.75 (1.57)
	Urticaria	Skin & subcutaneous tissue disorders	2.19 (1.89–2.53)	1.10 (0.89)
	Injection-site warmth	General disorders & administration site conditions	1.82 (1.54–2.16)	0.85 (0.60)
FLUMIST	Expired drug administered*	Injury & procedural complications	34.97 (30.49–40.10)	4.84 (4.64)
	Influenza-like illness	Infections & infestations	18.15 (14.23–23.15)	4.04 (3.68)
	Nasal congestion	Respiratory, thoracic and mediastinal disorders	9.09 (6.98–11.85)	3.11 (2.73)
	Rhinorrhoea	Respiratory disorders	8.61 (7.16–10.35)	3.02 (2.75)
FLUZONE	Peripheral swelling	General disorders & administration site conditions	5.61 (4.91–6.40)	2.36 (2.17)
	Extra dose administered	Injury & procedural complications	2.89 (2.52–3.32)	1.48 (1.28)
	Skin warm	Skin & subcutaneous tissue disorders	2.83 (2.50–3.21)	1.45 (1.26)
	Injection-site warmth	General disorders & administration site conditions	2.40 (2.19–2.64)	1.21 (1.07)
	Injection-site swelling	General disorders & administration site conditions	2.25 (2.09–2.43)	1.11 (1.00)

Disproportionality was assessed using reporting odds ratios (RORs) and information component lower bounds (IC025). ROR: A measure of the strength of disproportionality between a specific vaccine and adverse event; IC025: The lower bound of the 95% credibility interval of the Information Component, a Bayesian disproportionality metric. Only preferred terms with statistically significant signals (ROR lower 95% CI >1.0 and IC025>0) are presented.

*Denotes that “Expired drug administered” is a vaccine handling or procedural error, not a typical adverse event due to the vaccine's pharmacological action.

**Table 3:** Summary of rare adverse events following immunization (AEFIs) with quadrivalent influenza vaccines in pediatric populations.

AEFI	Total cases	Gender distribution	ROR (95% CI)	IC (IC025)	Median Time-to-Onset (IQR)	Afluria-IIV4	Fluarix-IIV4	Fluzone-IIV4	FluLaval-IIV4	Flucelvax-ccIIV4	FluMist-LAIV4
Guillain-Barre Syndrome	42	F: 24 (57.1%), M: 18 (42.9%)	1.71 (1.25–2.35)	0.73 (0.28)	14.5 days (4.5–32)	3	7	16	4	0	6
Myelitis Transverse	8	F: 4 (50.0%), M: 4 (50.0%)	1.18 (0.58–2.39)	0.22 (−0.76)	16.5 days (5–28.8)	0	0	4	–	1	1
Bell's Palsy	6	F: 3 (50.0%), M: 3 (50.0%)	1.00 (0.44–2.26)	0.00 (−1.12)	3.5 days (1–39.8)	1	1	1	3	0	0
Pericarditis	3	M: 3 (100%)	0.18 (0.06–0.56)	−2.42 (−3.87)	9 days (9–9)	–	–	2	–	–	–
Anaphylaxis Treatment	1	F: 1 (100%)	1.00 (0.44–2.26)	1.42 (−0.81)	1 day (1–1)	–	–	1	–	–	–
Myopericarditis	1	M: 1 (100%)	0.55 (0.08–3.99)	−0.84 (−2.92)	-	–	–	–	–	–	–

This table presents selected rare Adverse Events Following Immunization (AEFIs) identified in pediatric VAERS data (2013–2024), including vaccine-specific counts for each FDA-approved quadrivalent influenza vaccine (QIV). Events like Guillain–Barré Syndrome and myelitis among the most frequently observed rare AEFIs. Given the low event counts, findings are descriptive in nature and do not imply causality.

For context, the background incidence of Guillain-Barré syndrome in children is estimated at approximately 0.3–1.3 per 100,000 person-years; thus, while GBS met both disproportionality thresholds (ROR and IC), this likely reflects reporting enrichment rather than a true increase in clinical incidence (21, 22, 28, 29).

### 3.5 Temporal trends

**Figure 1:**
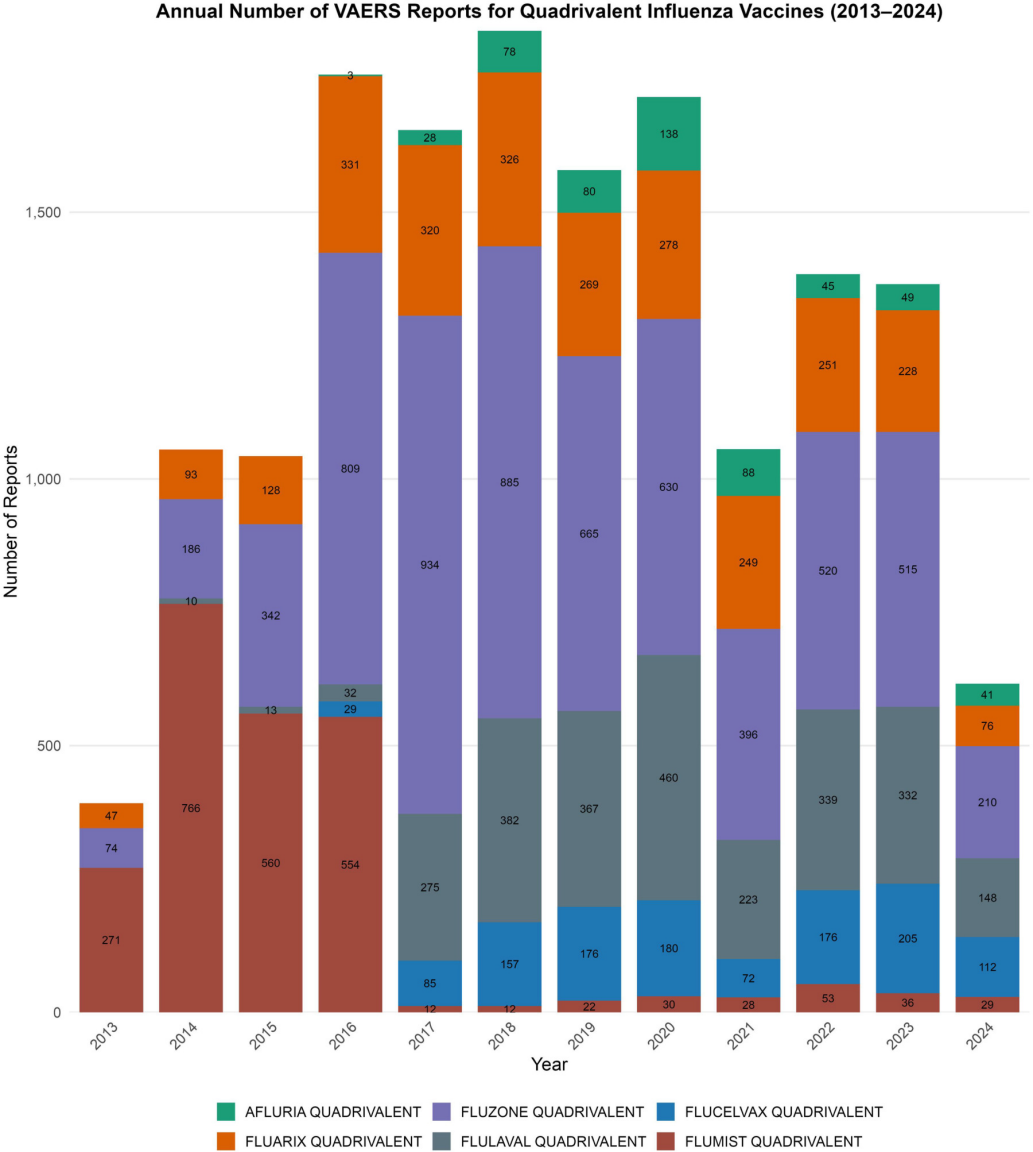
Annual pediatric VAERS reports by quadrivalent influenza vaccine formulation, 2013–2024. Stacked bar chart displaying the number of vaccine-specific adverse event reports submitted to VAERS each year for children under 18 years of age. Vaccines included Fluzone, Fluarix, FluLaval, FluMist, Flucelvax, and Afluria. A peak in overall reporting was observed in 2018, followed by a substantial decline during the COVID-19 pandemic period (2021–2024). Differences in volume across formulations and timeframes reflect variations in usage, market share, and potential reporting behavior.

Annual reporting volume peaked in 2018 with 1,840 reports, followed by a significant reduction ranging from 24.8% to 66.5% during 2021–2024, coinciding with disruptions related to the COVID-19 pandemic. Figure 1 illustrates the annual pediatric VAERS reports by quadrivalent influenza vaccine formulation from 2013 to 2024, clearly demonstrating the impact of the pandemic on reporting patterns.

Among the inactivated vaccines, the median time to adverse event onset was 0 days (IQR: 0–1 day), with 75% of events occurring within 24 h post-vaccination. FluMist demonstrated a slightly broader onset range (IQR: 0–2 days).

To assess potential underreporting during the COVID-19 pandemic, we compared pediatric VAERS reporting volume with national influenza vaccination coverage estimates. Coverage among children aged 6 months-17 years declined from 63.7% (2019–20) to 58.6% (2020–21),and remained relatively stable at 57.8% (2021–22) and 57.4% (2022–23), with a further decline to 55.4% (2023–24) (30–33).By contrast, VAERS reports dropped by 42%–66.5%, a disproportionate decline relative to vaccine uptake, supporting the inference that pandemic-related factors-such as reduced healthcare access, shifting reporting priorities, and system burden-likely contributed to underreporting rather than a true reduction in adverse event incidence.

### 3.6 Comprehensive safety signal analysis

**Figure 2:**
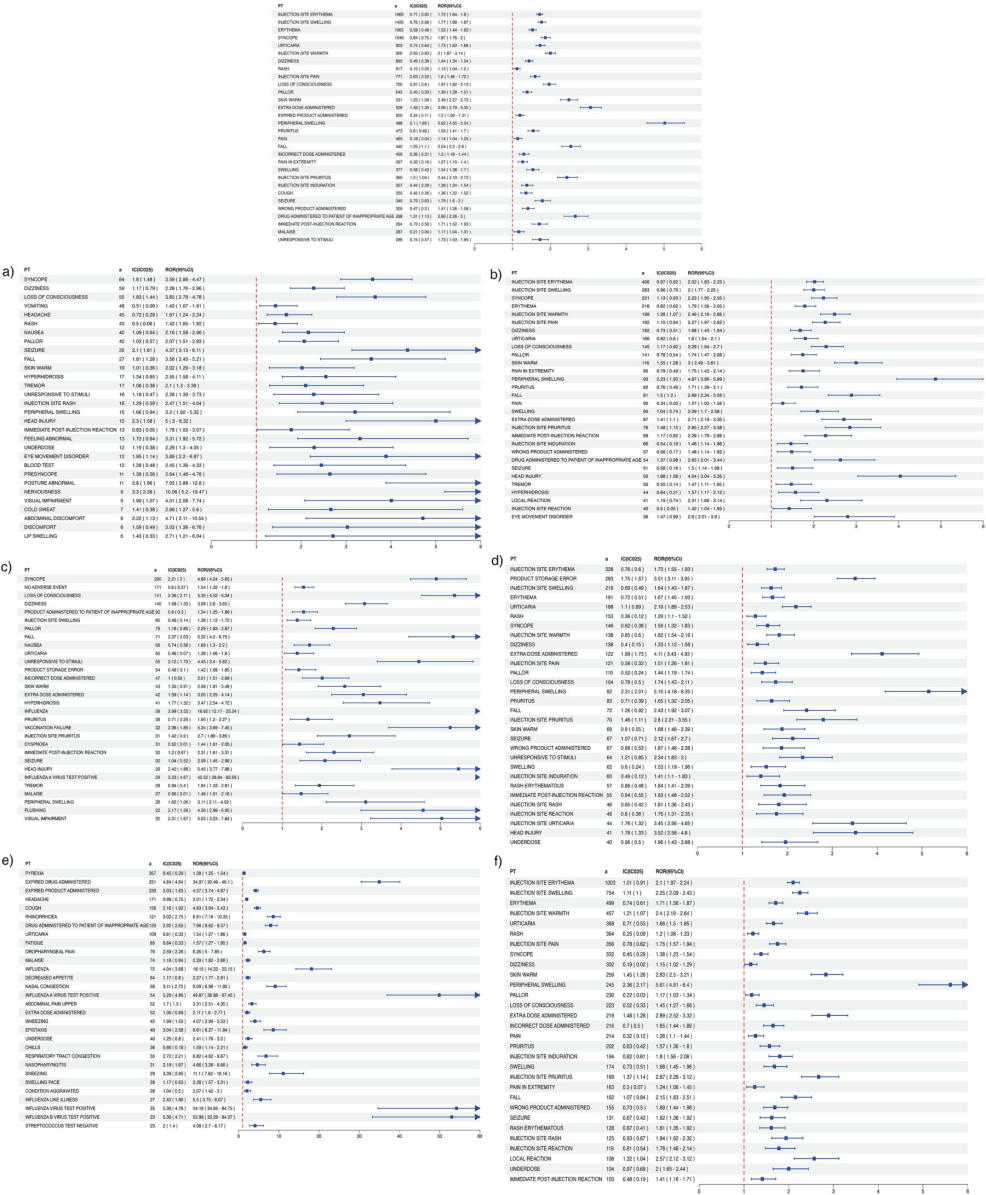
Disproportionality analysis of the 30 most frequently reported adverse events following quadrivalent influenza vaccination in children. Forest plot showing reporting odds ratios (ROR) and Bayesian Information Components (IC) with 95% confidence intervals for the 30 most frequently reported adverse events in VAERS (2013–2024), following administration of six FDA-approved quadrivalent influenza vaccines in children. ROR: A measure of the strength of disproportionality between a specific vaccine and adverse event. IC025: The lower bound of the 95% credibility interval of the Information Component, a Bayesian disproportionality metric. Significant signals were defined by ROR lower bound >1.0 and IC025>0. Blue squares represent point estimates; horizontal lines denote 95% confidence intervals. The red dashed line indicates the null value (ROR = 1.0). Arrowheads reflect intervals exceeding the plot range. Subfigures: **(a)** Afluria **(b)** Fluarix **(c)** Flucelvax **(d)** FluLaval **(e)** FluMist **(f)** Fluzone.

Figure 2 presents disproportionality analyses for the 30 most frequently reported adverse events following quadrivalent influenza vaccination in children. The forest plots demonstrate vaccine-specific reporting odds ratios (ROR) and Bayesian information components (IC) with 95% confidence intervals for each of the six FDA-approved QIVs. Significant signals were defined by ROR lower bound >1.0 and IC025>0, with notable variations observed across different vaccine formulations.

**Figure 3:**
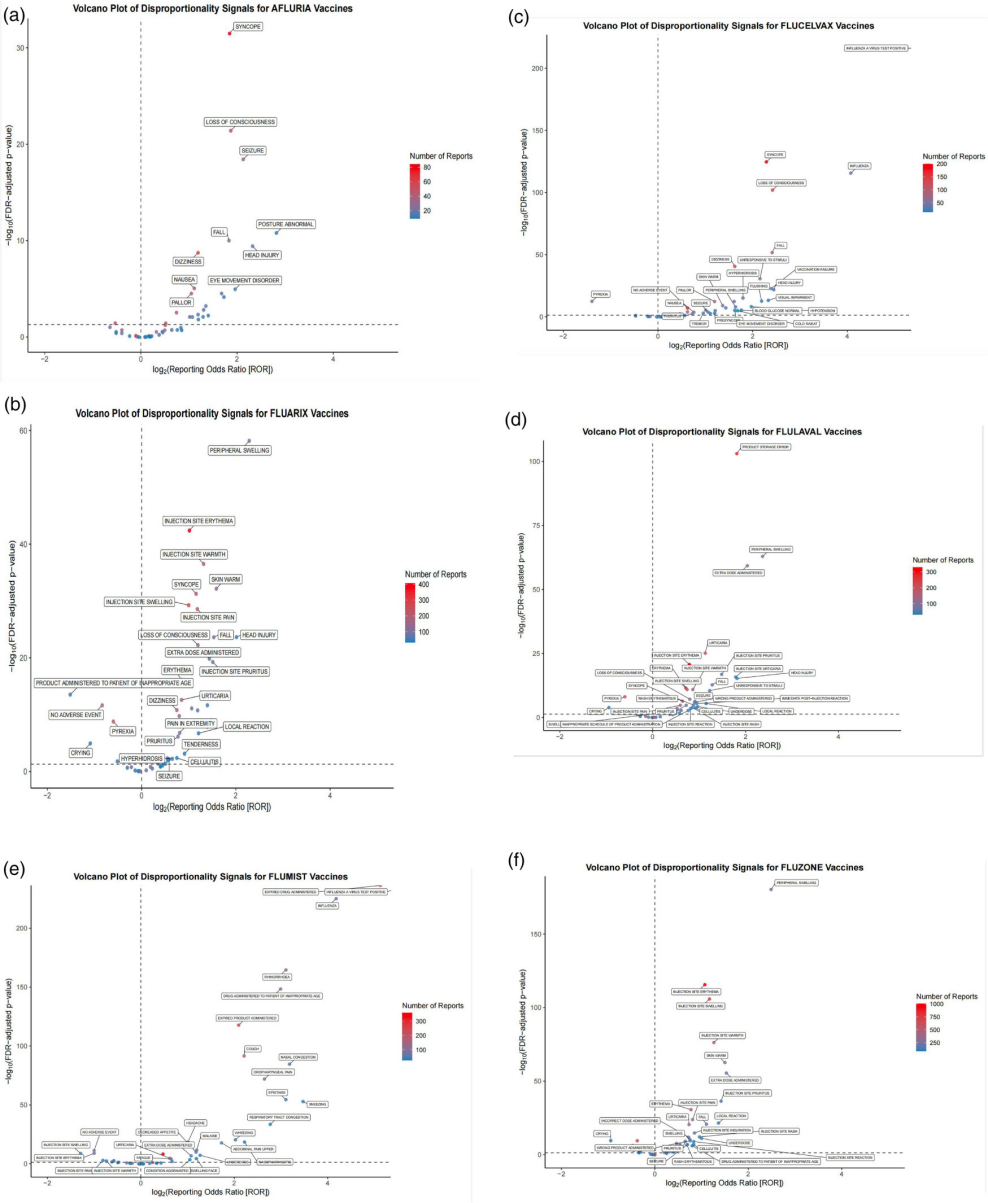
Volcano plots of disproportionality signals for quadrivalent influenza vaccines in pediatric populations. Disproportionality analysis of adverse event reports submitted to VAERS (2013–2024) for six FDA-approved quadrivalent influenza vaccines administered to children. Each volcano plot displays individual MedDRA preferred terms, with the *x*-axis representing the log_2_-transformed reporting odds ratio (ROR) and the *y*-axis indicating the -log_10_ of the false discovery rate (FDR)-adjusted *p*-value. Terms plotted further to the right and higher on the graph represent stronger disproportionality signals. The number of reports is indicated by dot size. Subfigures: **(a)** Afluria, **(b)** Fluarix, **(c)** Flucelvax, **(d)** FluLaval, **(e)** FluMist, **(f)** Fluzone.

To further characterize the safety signal landscape, Figure 3 displays volcano plots of disproportionality signals for each QIV. These plots effectively visualize the relationship between statistical significance and effect size, with each point representing an individual MedDRA preferred term. The *x*-axis represents the log_2_-transformed reporting odds ratio (ROR), while the *y*-axis indicates the log_10_ of the false discovery rate (FDR)-adjusted *p*-value. Terms positioned further to the right and higher on the graph represent stronger disproportionality signals, with dot size indicating the number of reports.

**Figure 4:**
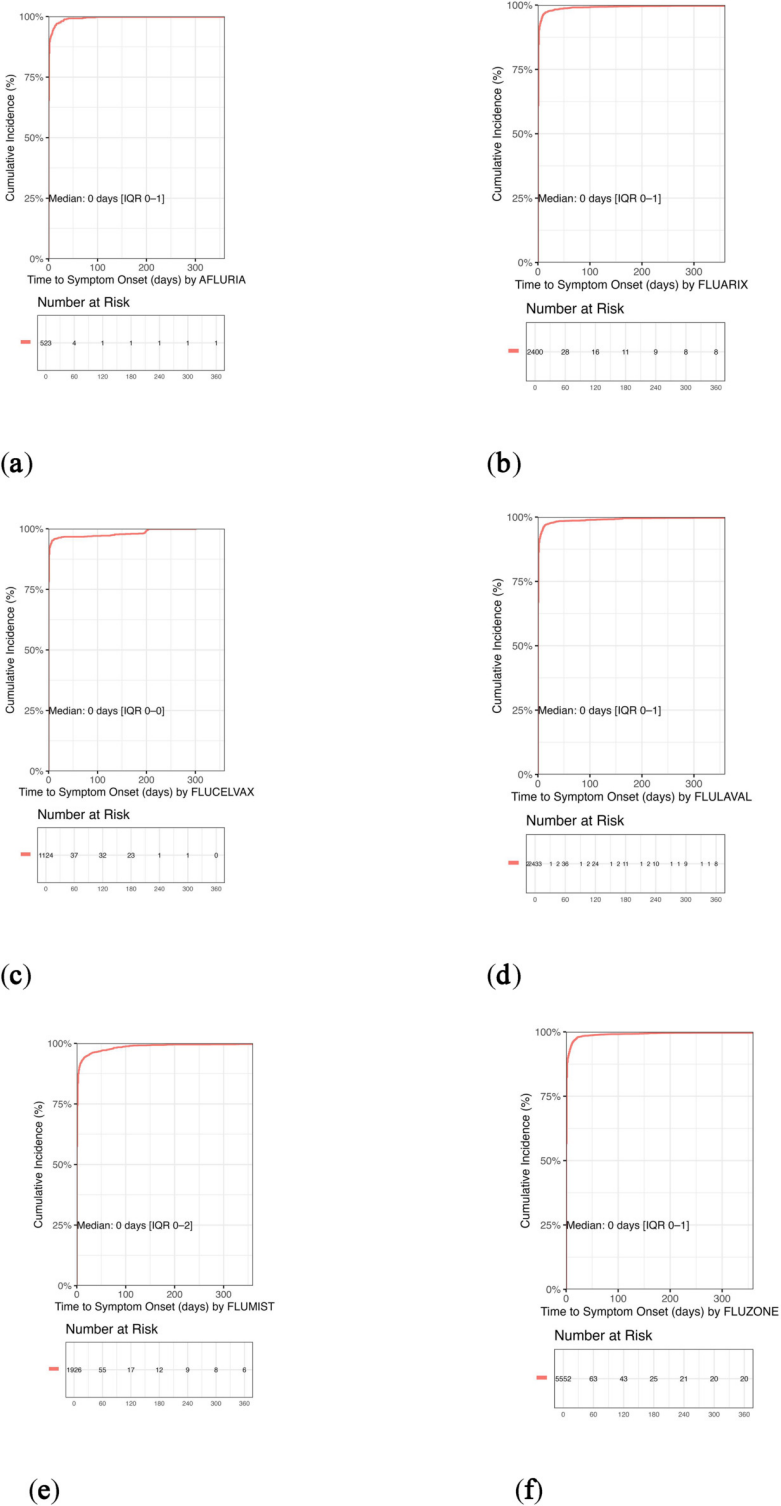
Kaplan–Meier curves of cumulative incidence of adverse events following quadrivalent influenza vaccination in pediatric populations. Kaplan–Meier curves depict the cumulative incidence of adverse events over time for each quadrivalent influenza vaccine formulation based on pediatric VAERS data (2013–2024). Across all vaccines, the median onset was 0 days, indicating an acute response profile. Over 95% of adverse events occurred within the first 7 days, with most reported on the day of vaccination. **(a)** Afluria: Median onset 0 days (IQR: 0–1); approximately 95% of events occurred on day 0. **(b)** Fluarix: Median onset 0 days (IQR: 0–1); similar to other inactivated formulations. **(c)** Flucelvax: Median onset 0 days (IQR: 0–0); exhibited the most rapid onset profile. **(d)** FluLaval: Median onset 0 days (IQR: 0–1); consistent with inactivated vaccine trends. **(e)** FluMist: Median onset 0 days (IQR: 0–2); slightly broader distribution typical of live attenuated vaccines. **(f)** Fluzone: Median onset 0 days (IQR: 0–1); rapid onset maintained despite higher report volume. The rapid onset patterns across all vaccines support the acute nature of most reported events and align with known immunization response timelines.

To further explore formulation-specific safety profiles, we conducted a sensitivity analysis stratified by vaccine type-inactivated influenza vaccines (IIV4) vs. live attenuated influenza vaccine (LAIV4). This analysis integrated findings from Tables 1, 2 and Figures 2–4. IIV4s (Fluzone, Fluarix, FluLaval, Afluria, and Flucelvax) collectively accounted for 84.6% of reports and were more frequently associated with neurological and injection-site events, such as syncope, seizures, and injection-site erythema. In contrast, LAIV4 (FluMist) was more commonly linked to upper respiratory symptoms including rhinorrhea and nasal congestion. Time-to-onset patterns were broadly similar, with most events occurring within the first day post-vaccination, though LAIV4 showed a slightly wider onset range (IQR: 0–2 days). These trends are consistent with known reactogenicity profiles and vaccine administration routes.

### 3.7 Time to symptom onset patterns

Figure 4 presents Kaplan–Meier curves depicting the cumulative incidence of adverse events over time for each quadrivalent influenza vaccine formulation. Across all vaccines, the median onset was 0 days, indicating an acute response profile. Over 95% of adverse events occurred within the first 7 days, with most reported on the day of vaccination. These rapid onset patterns support the acute nature of most reported events and align with known immunization response timelines.

## 4 Discussion

### 4.1 Principal findings

This large-scale post-marketing surveillance study provides robust evidence supporting the overall safety of quadrivalent influenza vaccines (QIVs) in pediatric populations. The observed 5.29% rate of serious adverse events (SAEs) reflects the inherent characteristics of passive surveillance systems. Large-scale active surveillance studies conducted through the Vaccine Safety Datalink demonstrate substantially lower rates of confirmed serious adverse events, with one study of over 590,000 live attenuated vaccine doses finding only rare confirmed cases of anaphylaxis (1.7 per million doses) and syncope (8.5 per million doses) (34), while another study of over 91,000 inactivated vaccine doses in young children found no evidence of serious medically attended events (35). However, interpretation must consider the inherent limitations of passive surveillance systems, which typically capture only 1%–10% of actual adverse events and are more likely to detect serious outcomes (36).

### 4.2 Guillain-Barré syndrome risk-benefit assessment

Among the rare adverse events assessed, Guillain-Barré syndrome (GBS) was the only one that met both signal detection thresholds (ROR=1.71), consistent with selected epidemiological studies (37). While some meta-analyses have found no confirmed risk of vaccine-associated GBS (38), CDC surveillance has reported modest relative risk increases within expected post-vaccination intervals (17). The median onset of 14.5 days is biologically plausible and supports a potential immune-mediated mechanism.

Importantly, the absolute risk of GBS following QIV administration remains exceedingly low. Even at the upper limit of the confidence interval, the estimated excess risk is 1–2 additional cases per million doses. This must be balanced against the substantially higher GBS incidence following natural influenza infection, which is estimated to be 4–7 times greater (37, 39). Recent pediatric surveillance data indicate a background incidence of Guillain-Barré syndrome of approximately 0.34–0.69 per 100,000 person-years in children under 16 years (22). Therefore, although GBS met both disproportionality thresholds (ROR and IC), this signal most likely reflects nhanced reporting rather than a clinically meaningful increase in incidence.

### 4.3 Age-dependent reactogenicity

The elevated SAE rates observed in children aged 6 months to 5 years are clinically noteworthy. This group exhibited 2- to 4-fold higher SAE rates compared to adolescents, which may reflect age-related differences in immune response and heightened parental or provider vigilance in reporting (40, 41). The immature immune system characteristics in early childhood, including differences in innate and adaptive immune responses, may contribute to varying reactogenicity profiles across pediatric age groups (40, 42).

### 4.4 Platform-specific safety profiles

Flucelvax demonstrated the lowest SAE rate (2.35%), suggesting potential safety advantages associated with cell culture-based platforms, including the absence of egg-adaptive mutations (43) and possibly lower innate immunogenicity due to production processes. Conversely, the elevated frequency of neurological adverse events associated with Afluria is noteworthy, especially given prior reports linking this formulation to febrile seizures (44, 45). These findings support the hypothesis that manufacturing methods and antigenic composition may influence vaccine reactogenicity.

### 4.5 Impact of the COVID-19 pandemic

The marked decline in annual adverse event reporting (24.8%–66.5%) during 2021–2024 likely reflects the multifactorial consequences of the COVID-19 pandemic, including reduced pediatric immunization visits, shifts in healthcare delivery, and reporting fatigue among healthcare providers (24, 46, 47). Additionally, changes in public health messaging, vaccine formulation preferences, and seasonal uptake may have further confounded reporting trends.

### 4.6 Public health implications

These findings may inform tailored vaccine recommendations, particularly in pediatric subgroups. For instance, cell-based QIVs may offer preferable safety profiles for immunocompromised children or those with a history of seizures (48, 49). Continued surveillance and stratified safety assessments are essential for optimizing vaccine selection across diverse populations (50).

### 4.7 Study limitations

This study is subject to several limitations inherent to the VAERS system, including underreporting, reporting and selection bias, absence of denominator data, and the inability to determine causality without an unvaccinated comparator group. These constraints highlight the importance of corroborating findings with active surveillance systems and well-controlled epidemiological studies.

## 5 Conclusions

This 12-year analysis of 15,458 pediatric reports from the VAERS database provides compelling evidence affirming the overall safety of FDA-licensed quadrivalent influenza vaccines (QIVs) in children. The predominance of non-serious, self-limiting adverse events and the relatively low rate of serious outcomes underscore a favorable benefit-risk profile.

Although Guillain-Barré syndrome (GBS) met signal detection thresholds, its absolute incidence remained exceedingly low. These findings reinforce the safety of QIVs and support continued adherence to national influenza vaccination guidelines.

Importantly, formulation-specific differencessuch as the notably lower SAE rate observed with Flucelvax-suggest that cell-based QIVs may be preferable in younger or medically vulnerable subgroups, although further research is warranted. Age-related variation in reactogenicity also underscores the need for targeted risk communication and vigilant monitoring, particularly in children aged 6 months–5 years.

Given the observed drop in reporting during the COVID-19 pandemic, future efforts should integrate active surveillance systems such as the Vaccine Safety Datalink (VSD) to complement passive data sources and ensure timely signal detection. Pediatric healthcare providers can remain confident in recommending annual influenza vaccination, while supporting ongoing safety evaluation through systematic reporting and data sharing.

## Data Availability

The raw data supporting the conclusions of this article will be made available by the authors, without undue reservation.
